# Comparison of Risk Factors Between Syndesmotic Screws With and Without Breakage

**DOI:** 10.7759/cureus.48320

**Published:** 2023-11-05

**Authors:** Josh W Vander Maten, Christopher G Sanford, Matthew McCracken, Elisabeth Sohn, Alyssa Lange, Eric Zhang, Logan Roebke, Jiayong Liu

**Affiliations:** 1 Department of Orthopedic Surgery, The University of Toledo Medical Center, Toledo, USA

**Keywords:** risk factor, screw breakage, syndesmotic screw, syndesmotic disruption, syndesmosis, ankle

## Abstract

Background

Screw fixation continues to be a commonly used treatment for syndesmotic disruption; however, screw breakage remains a complication post-fixation. Despite this complication, investigation on the variability of surgical placement in conjunction with syndesmotic screw characteristics affecting breakage has not been fully elucidated. The purpose of this study is to compare patients with syndesmotic screw breakage versus those with intact screws based on surgically controlled variables.

Methods

A total of 176 patients and 260 syndesmotic screws were included in the study, 88 patients each with and without broken syndesmotic screws. A retrospective analysis of patients who underwent syndesmotic screw fixation was performed. Patients with syndesmotic screw breakage were compared to those with intact screws. Screw width and length, the number of screws used, fracture type, and the number of cortices for fixation were all collected. Further analysis included radiographic measurement of syndesmotic screw angle and height of placement above the tibial plafond.

Results

Decreased screw width, increased number of screws used, and younger age were all associated with increased rates of screw breakage (p < .001, p = .019, p = 0.020). No statistical difference was appreciated between groups based on screw length, number of cortices used, or angle relative to the tibial plafond (p = .2432, p = .4699, p = .9233).

Conclusion

Higher placement of syndesmotic screws above the tibiotalar joint, specifically greater than 20 mm above the tibial plafond, increases the screw breakage rate. Decreased screw width, increasing numbers of screws used, and younger age were all also associated with increased rates of screw breakage. No difference was appreciated based on the screw angle relative to the tibial plafond.

## Introduction

Screws are frequently used for the fixation of syndesmosis disruption. The fixation of syndesmosis disruption with screws remains a frequent and efficacious treatment modality [1,2]. While other techniques, including suture button devices, bioabsorbable screws, and directed ligament repair may be used, fixation with screws remains a commonly employed technique [1-12]. Metal screws have been used and studied for decades and when compared to other fixation methods, syndesmotic screws are relatively low-cost [2,3,5,13]. However, there is still some controversy regarding routine removal, as doing so can impact patient outcomes in different ways [7-9]. As such, many patients may retain their screws, which can predispose them to screw breakage and/or discomfort [10].

Breakage of screws is a well-known and common complication seen in many patients following syndesmotic fixation with metal screws. Although other complications, such as mal-reduction or the need for additional surgery, may occur, screw breakage remains the most commonly reported [6,11,14]. Depending on the location of the screw breakage, patients may experience varying functional outcomes. For instance, breakage within an intraosseous location, defined as within the tibia or fibula, may lead to increased pain, subsequent removal, and associated complications [10,15-16]. This increased pain may be caused by repetitive physiologic rotation of the tibia and fibula, which can lead to bony erosion [9]. In some cases, a device has been proposed and studied to encourage breakage within the clear space, defined as the space between the tibia and fibula, in a small cohort of patients [14]. While the location of screw breakage along the length of the screw remains unpredictable, intraosseous breakage may occur more frequently [10]. The placement of the screw with regard to the tibiotalar joint may also influence the rate of intraosseous breakage. Screws placed closer to the tibiotalar joint may protect against intraosseous breakage while those placed >20 mm above the plafond may increase the risk of clear space breakage [10]. However, the impact of the surgical placement variables in conjunction with screw characteristics affecting the rate of screw breakage in patients with syndesmotic screw fixation has not been thoroughly investigated. Therefore, the objective of this study is to compare patients with syndesmotic screw breakage and patients with intact screws based on surgical placement variables in conjunction with screw characteristics. The study aims to provide a better understanding of the effect of surgical placement variability on screw breakage.

## Materials and methods

Following institutional review board approval by the University of Toledo Human Research Protection Program Biomedical IRB, a retrospective analysis of patients who underwent syndesmotic screw fixation from 2008 to 2020 was performed. The majority of patients underwent syndesmotic fixation in conjunction with an ankle fracture resulting in syndesmotic disruption. Patients underwent syndesmotic fixation at either the time of primary open reduction and internal fixation (ORIF) surgery or shortly after in the case of severe trauma requiring periods of external fixation. Only a few patients underwent syndesmotic fixation for isolated syndesmotic diastasis secondary to ankle trauma without resulting fracture or a Maisonneuve fracture. A total of 176 patients and 260 syndesmotic screws were included in the study. Each group comprised 88 patients. Ninety-seven patients with skeletally mature bone were found to have syndesmotic screw breakage, of which 88 met the inclusion criteria. All patients received syndesmosis screw fixation as well as plate and screw fixation for distal fibular fractures. A clear postoperative radiograph demonstrating screw breakage within the tibia, fibula, or clear space was required for inclusion. All syndesmotic fixation using methods other than screw fixation were excluded.

**Figure 1:**
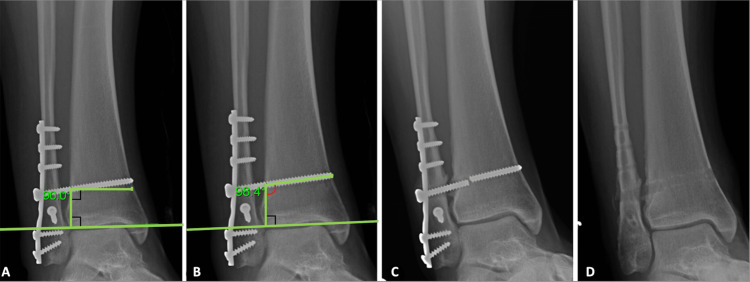
Series of radiographs All radiographs are from a mortise view. The patient was a 20-year-old female with a BMI of 33.7 who suffered a distal fibula fracture with syndesmotic instability. Surgical fixation was done using a plate and screw construct with a single 4.5 mm syndesmotic screw. Radiograph demonstrating a 90-degree angle with tibial plafond joint reference used to measure screw angle (A). The screw was placed at a 98.4-degree angle in this patient (B). After 57 weeks, a single syndesmotic screw broken within the tibia was seen on follow-up imaging (C). Due to persistent and worsening pain, all hardware, including the syndesmotic screw, was removed (D). No further complications were seen following removal.

**Figure 2:**
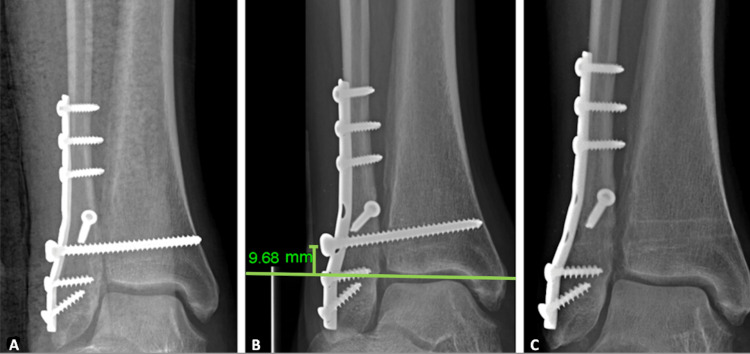
Full radiograph sequence All radiographs are from a mortise view. Images of a 53-year-old male with a BMI of 66 are shown. Postoperative radiographs using a fibular plate and a single 4.5 mm syndesmotic screw are shown (A). A radiograph demonstrating the measurement of the height of screw placement of 9.68 mm above the tibiotalar joint (B). The syndesmotic screw was removed 15 weeks later due to persistent pain and discomfort (C).

Following identification and complete data collection of the breakage group, an additional 88 patients without syndesmotic screw breakage were identified to serve as the control group. Patients in this group were chosen at random within the same time frame as the breakage group. Basic patient demographic data were collected, including age, gender, and body mass index (BMI). Screw variable analysis included screw width and length, the number of screws used, ankle fracture type, and the number of cortices used for fixation. The surgical variable analysis included radiographic measurement of the syndesmotic screw angle relative to the tibiotalar joint and height of placement above the tibiotalar joint. A series of radiographs and a demonstration of the measurement of screw placement above the tibiotalar joint from a patient with syndesmotic screw breakage is shown in Figures 1A-1D. All measurements were done using our institution’s picture archiving and communication system (PACS). Of note, all screw angles relative to the tibial plafond are relative to a 90-degree angle used as a reference point. The full radiograph sequence and screw angle measurement relative to the tibial plafond are shown in Figures 2A-2C. Screws within the breakage group and the intact control group were measured using the first mortise radiograph following surgical fixation. Intact screws in patients who had at least one broken screw were also measured for separate comparison. Independent t-tests, chi-square analysis, and Fischer exact tests were used to compare means between groups.

Postoperative management included non-weight-bearing status for six weeks with regular physical therapy. A gradual progression to full weight-bearing was then employed with full weight-bearing status occurring no earlier than 12 weeks. Patients were typically seen for clinical follow-up at two, four, six, and 12 weeks postoperatively. All operative decisions, including screw size, screw number, amount of cortices screws traversed, screw placement angle, and screw placement height, were made by our institutional surgeons, who are all well-equipped to treat ankle fractures, using their best clinical judgment. Diagnosis of syndesmotic disruption was made using radiographic evidence, including tibiofibular overlap, tibiofibular clear space talar tilt, and intraoperative stress testing.

## Results

The breakage group had a total of 121 syndesmotic screws while the control group had 139. The breakage group was comprised of 45 males (51.1%) and 43 females (48.9%) with an average age of 38.88 (SD + 15.61). The control group consisted of 47 males (53.4%) and 41 females (46.6%) with an average age of 45.12 (SD + 16.69). Patients with broken screws were found to be significantly younger than those with intact screws (p = .0148). An average BMI of 33.55 (SD + 9.818) was seen in the breakage group and 31.58 (SD + 8.15) in the control group (p = .1346) (Table 1).

**Table 1:** Patient and screw characteristics

Variable	Broken	Intact	p-value
Patients (n)	88	88	
Screws (#)	121	139	
Patient characteristics (averages)			
Age	38.88	45.12	0.0148
Gender, n (%)			
Male	45 (51.1)	47 (53.4)	0.880
Female	43 (48.9)	41 (46.6)	
BMI (kg/m2)	33.55	31.58	0.1346
Screw characteristics (averages)			
Number of screws used, n (%)	1.89	1.55	
1 screw	29 (33.0)	42 (47.7)	0.019488
2 screws	46 (52.3)	43 (48.9)	
> 2 screws	12 (13.6)	3 (3.4)	
Screw length (mm)	52.88	53.79	0.2432
Screw width (mm)	3.67	4.06	< .001>
Number of cortices	3.45	3.50	0.4699
Screw angle (degrees), range	94.82 (55.6 - 111.0)	94.67 (66.0 - 121.9)	0.9233
Screw height (mm), range	20.39 (2.19 - 50.49)	16.72 (-5.41 - 37.85)	0.0033
Screws broken, n	1.53	N/A	

When compared to the breakage group, more patients within the control group used a single syndesmotic screw (n = 29, n = 42). A nearly identical number of patients within each group used two syndesmotic screws (n = 46, n = 43) while more patients within the breakage group used > 2 syndesmosis screws when compared to the control group (n = 12, n = 3). There was a significant association between screw breakage and an increased number of screws used (p = .019). The average screw width in the breakage group was 3.67 (SD =.38) and 4.06 (SD =0.04) in the intact group (p =< .001). No statistical difference was appreciated between groups based on screw length (p = .2432) or the number of cortices used (p = .4699) (Table 1).

Mean screw placement above the tibiotalar joint was 20.39 mm (SD =10.39) in the breakage group and 16.75 mm (SD = 9.28) (p = .0033) in the control group. No statistical difference was appreciated between groups based on the angle relative to the tibial plafond (p = .9233) (Table 1).

Following stratified analysis based on distribution, no difference was appreciated between groups based on screw angle (p = .099). A significant difference was appreciated when comparing screw placement (p = .022). Fifty-two percent of patients within the breakage group had screws placed > 20 mm above the tibial plafond compared to 34.5% in the control group. Fracture type showed no difference between groups (p = .272) (Table 2).

**Table 2:** Screw angle, screw placement, and fracture type analysis

	Broken, screw # (%)	Intact, screw # (%)	Pearson chi-square
Screw angle (degrees)			
<82	13, (10.7)	8, (5.8)	0.099
82 - 94	46 (38)	72 (51.8)	
94 - 108	46 (38)	47 (33.8)	
>108	16 (13.2)	12 (8.6)	
Screw placement (mm)			
<10	18 (14.8)	40 (28.8)	0.022
10 - 20	42 (34.7)	52 (37.4)	
20 - 30	41 (33.8)	37 (26.6)	
>31	20 (16.5)	10 (7.1)	
Fracture type			
Unimalleolar	53 (43.8)	66 (47.5)	0.272
Bimalleolar	31 (25.6)	45 (32.4)	
Trimalleolar	26 (21.5)	16 (11.5)	
Maisonneuve	2 (1.7)	1 (0.7)	
Syndesmosis	9 (7.4)	11 (7.9)	

Finally, when comparing intact screws against broken screws in patients with at least one broken screw and one intact screw, no statistically significant difference was appreciated based on any measured variable (Table 3).

**Table 3:** Non-broken and broken screws in patients with at least one broken screw

	Patients (n)	Screws (#)	Male, n (%)	Female, n (%)	Number of screws used, n	2 screws, n (%)	> 2 screws, n (%)	Screw length (mm)	Screw width (mm)	Screw angle (degrees), range	Screw height (mm), range
Broken	39	48	21 (53.85)	18 (46.15)	2.33	29 (47.4)	10 (25.6)	53.04	3.67	96.90 (55.6 - 111.0)	19.87 (2.19 - 50.49)
Intact		40						52.34	3.625	95.77 (68.2 - 137)	19.69 (-4.98 - 56.13)
p-value								0.5913	0.5258	0.6667	0.9396

## Discussion

Historically, syndesmotic screw breakage has been thought to be of little significance. Recently, evidence has been presented suggesting syndesmotic screw breakage can lead to increased pain depending on the breakage location [10]. However, few studies have critically examined the angle and placement of the syndesmotic screw in relation to the tibiotalar joint, along with the characteristics of the screw itself. Furthermore, no study, to our knowledge, has performed a comparative analysis between patients with and without syndesmotic screw breakage using the variables presented here.

In this retrospective comparative study, we found that with increasing height of placement above the tibiotalar joint, there is a greater likelihood of syndesmotic screw breakage. Specifically, placement of the screw more than 20 mm above the tibial plafond is associated with a significantly increased risk of screw breakage. Additionally, following the stratification of these findings, the differences between groups persist. Decreased screw width, increasing numbers of screws used, and younger age were all related to an increased rate of screw breakage. While some may expect that increasing the number of screws would allow for some degree of load distribution, it was uncovered that the number of screws used is significantly associated with an increased risk of screw breakage. However, each screw could also simply represent an additional opportunity for screw breakage, and patients with multiple screws placed would inherently be at increased risk for screw breakage. Notably, the screw angle relative to the tibial plafond did not demonstrate any significant association with the breakage rate. However, it is important to note only anteroposterior (AP) and mortise view X-rays were available for analysis, limiting our analysis of screw angle to the coronal plane. Due to the nature of this retrospective study, the inability to examine other planes may have limited our ability to detect a significant association between the angle of screw placement and the risk of screw breakage.

A recent study by Ibrahim et al. found that in patients with syndesmotic screw breakage, screws placed greater than 20 mm above the tibial plafond were at increased risk for breakage within an intraosseous location when compared to clear space breakage. However, no control group for comparison was included and conclusions were made comparing only broken screws based on breakage location [10].

Many studies have presented conflicting conclusions on the optimal height of syndesmotic screw placement. Optimal placement height has been suggested as 30-40 mm, 20 mm, and 1 cm above the tibial plafond [17-19]. Kukreti et al. performed a retrospective analysis and found no difference in clinical or radiographic outcomes between screws placed below 20 mm of the tibial plafond and those placed above [20]. Other studies have determined that syndesmotic screw placement has no clinically significant impact on patient outcomes. However, many of these studies are cadaveric or radiographic in nature [17,19,20]. Of the studies that do clinically examine patients, no study focuses on comparing patients with screw breakage versus those without [16,20]. Additionally, no study has attempted to use a retrospective comparative study to determine the risk of syndesmotic screw breakage based on the variables presented in the current study.

Many studies have examined screw failure based on screw width and the number of cortices used [21-26]. The consensus remains that the increased width of syndesmotic screws is protective against screw breakage. Here, we agree with these studies, as an increased average screw width within the control group was found to be protective against screw breakage. Previously, Mendelsohn et al. found that increased BMI was associated with an increased risk of screw failure [27]. In our cohort, we did not find any difference in BMI between groups. Remarkably, we found that younger patients were also at an increased risk of screw breakage. This could, in theory, correlate with increased activity levels, increased ability to partake in higher-intensity activities, and a higher necessity to return to early weight-bearing activity due to family or work concerns. In younger individuals, bone quality is higher and thus the screw cannot easily be cut out or pulled out together. Earlier weight bearing can transfer stress to these screws and thus lead to higher rates of breakage. We are of the opinion that proper postoperational management of weight-bearing can help prevent screw breakage in younger individuals.

Notably, we did attempt to compare non-broken screws against broken screws in patients that had at least one broken screw and at least one intact screw. Although 39 patients with 48 broken and 40 intact screws met these criteria, we did not appreciate any significant findings similar to those found in our initial comparison.

It is our opinion that surgeons should be mindful of the height of placement above the plafond when using syndesmotic screws. Further, younger patients should be counseled on the increased risk of screw breakage and counseled accordingly based on the surgeon’s experience as the clinical significance of screw breakage remains an active debate.

While our data uncovers many meaningful findings, there are still limitations to this retrospective study. Particularly, many of the patients did not receive computerized tomography (CT), which made analyzing screw angles within the sagittal plane difficult. Ideally, we would have included the angle of screw placement relative to the sagittal plane in our comparison, especially since the well-accepted 25-30 degrees of angulation have recently been called into question [28]. Future studies should attempt to incorporate this angle into the analysis that has been presented here.

## Conclusions

Higher placement of syndesmotic screws above the tibiotalar joint, specifically greater than 20 mm above the tibial plafond, increases the screw breakage rate. No difference was appreciated based on the screw angle relative to the tibial plafond in the coronal plane. In addition to placement, there are other factors that can contribute to an increased rate of screw breakage. For example, decreased screw width can make the screws more prone to breaking under stress. Using a higher number of screws to stabilize can also increase the likelihood of breakage. Finally, younger patients were also more prone to screw breakage. By taking these factors into account, there is a possibility of reduced risk of complications.
